# Clinic characteristics of psoriasis in China: a nationwide survey in over 12000 patients

**DOI:** 10.18632/oncotarget.18453

**Published:** 2017-06-12

**Authors:** Kun Chen, Gang Wang, Hongzhong Jin, Jinhua Xu, Xuejun Zhu, Min Zheng, Heng Gu

**Affiliations:** ^1^ Institute of Dermatology, Chinese Academy of Medical Sciences and Peking Union Medical College,Nanjing, Jiangsu, China; ^2^ Department of Dermatology, Xijing Hospital, Fourth Military Medical University, Xi'an, Shanxi, China; ^3^ Peking Union Medical Hospital, Chinese Academy of Medical Sciences and Peking Union Medical College, Peking, China; ^4^ Department of Dermatology, Huashan Hospital, Fudan University, Shanghai, China; ^5^ Department of Dermatology, Peking University First Hospital, Peking, China; ^6^ Department of Dermatology, Second Affiliated Hospital, Zhejiang University, School of Medicine, Hangzhou, Zhejiang, China

**Keywords:** psoriasis, epidemiology, phenotype, Chinese Han

## Abstract

Psoriasis is a worldwide chronic inflammatory disease, involving both skin and joints. In order to characterize psoriasis in Han Chinese population, we conducted this nationwide prospective and hospital based survey, in which 56 hospitals with departments of dermatology participated, located in 33 cities across China. A total of 12,031 outpatients with psoriasis were registered during 2009 to 2010, which the data was collected by standard questionnaires. The main data acquisition included demographics, family history, disease status and other comorbidities. Physical and dermatological examination, including body surface area (BSA) and psoriasis area severity index (PASI) were applied to evaluate the disease severity. Descriptive statistics, 2 tailed *t*-test and chi-square test were used appropriately for the statistical analysis. From the study, we found that the male and female ratio of the patients was 1.49:1. Mean age of onset was 30.2 ± 14.5 years for males and 27.1 ± 15.6 years for females (*P* < 0.05). Scalp was the most common onset site (52.8%), The mean PASI was 18.70 ± 10.01, indicating that most patients presenting at the hospitals had moderate-to-severe psoriasis and the majority was psoriasis vulgaris (96.5%). Among 12,031 patients, 23.1% had a family history of psoriasis,16.1% had comorbidities, and 29.9% had nail changes. The most important aggravation factor was season change (60.2%), followed by psychological stress (34.5%), and there significant differences between genders on trigger factors. In conclusion, this study characterizing psoriasis in Han Chinese population, could be used as basic data for future study.

## INTRODUCTION

Psoriasis is a chronic inflammatory skin disease with a strong hereditary component [1]. It is a heterogeneous disorder with considerable differences in age of onset, morphological appearance and location, severity, comorbidity, and response to established treatments [2]. For example, early onset is generally associated with HLA-Cw6, −B13 and −B57, while late onset is correlated with HLA-Cw2 and −B27 [3] Environmental factors also play important roles in the pathogenesis of psoriasis. Triggering factors like smoking, excessive alcohol consumption, infections, stress and certain medications were found to be important for the initial manifestation and/or disease flares. [4–7]. Chronic plaque psoriasis is the most common type. About 18% to 21% of patients with psoriasis have distinctive nail changes, and nearly11.2% to 14.3% develop seronegative inflammatory arthritis (psoriatic arthritis, PsA) in Asia [8, 9]. Furthermore, growing evidences have shown that psoriasis is associated with metabolic syndrome such as obesity, hypertension, diabetes, dyslipidemia, and psychiatric diseases [10, 11]. The prevalence of psoriasis varies throughout the world, apparently reflecting differences in genetic and environmental factors. The incidence, clinical features, and comorbidities have been widely studied in the United States and Europe countries [12–15]. In Western countries, the prevalence is 2–3%, across all ages [12]. Data on the epidemiology of psoriasis in Asian populations is scarce. It seems that the prevalence in Asian is lower than European and North Americans, basing on few published studies [12].

## RESULTS

### General characteristics of psoriasis in China

Of the 56 participating hospitals, 31 located in the North and 25 in the South (See Table 1). A total of 12,031 psoriasis patients were enrolled, 6005 of which in the North and 6026 in the south. The mean age of all patients was 39.42 ± 18.42 years old, and the ratio of male to female was 1.49:1. Among all patients, 23.1% had a positive family history of psoriasis (See Table 2).

**Table 1:** Distribution of hospitals

Cities	No.of hospitals	No.of cases	Location
Beijing	10	2513	North
Tianjing	2	473	North
Dalian	1	97	North
Xi'an	1	300	North
Haerbing	2	291	North
Changchun	2	200	North
Shenyang	2	329	North
Hohhot	1	49	North
Shijiazhuang	1	109	North
Zhengzhou	2	756	North
Taiyuan	3	763	North
Qingdao	1	80	North
Jinan	1	75	North
Lanzhou	1	38	North
Yinchuan	1	57	North
Xining	1	99	North
Urumqi	1	76	North
Hefei	1	72	South
Nanjing	1	1302	South
Wuxi	2	372	South
Hangzhou	3	289	South
Shanghai	5	1797	South
Fuzhou	1	12	South
Nanchang	1	24	South
Wuhan	1	493	South
Xianning	1	265	South
Changsha	1	75	South
Guangzhou	2	104	South
Guilin	1	18	South
Kunming	1	26	South
Chengdu	1	731	South
Chongqing	1	77	South
Haikou	1	69	South
Total	56	12031	

**Table 2:** Summary of demographics of patient population

Characteristics	*N* = 12031
**Sex *n* (%)**	
Male	7206 (59.9)
Female	4825 (40.1)
**Age, y**	
Min	1
Max	86
Mean (SD)	39.42 (18.4)
**Family history of psoriasis *n* (%)**	
Family history of psoriasis	2781 (23.1)
Relatives	*N* = 4995
First degree relatives	2323 (46.5)
Second degree relatives	1538 (30.8)
Third degree relatives	1134 (22.7)
**History of smoking and alcohol *n* (%)**	
Smoking history	4023 (33.4)
Quit smoking	936 (7.8)
Alcohol history	2524 (21.0)
Alcohol history > 10yrs	1281 (10.6)

**Figure 1:**
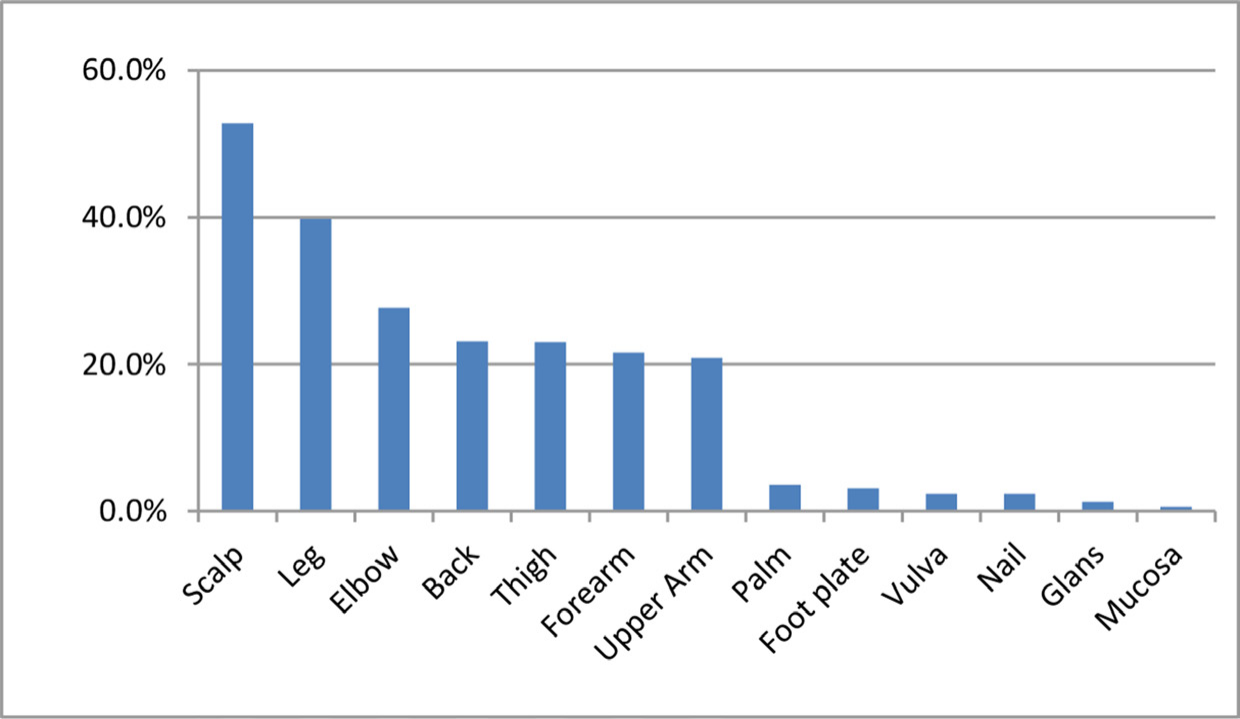
Onset position of patients

As we can see from Table 3, 89.7% of patients had received topical treatments and 79.8% had received systemic treatments. Scalp was the most common onset site (52.8%, See Figure 1), while legs were the most common distribution sites of lesions (77.7%, See Table 4). The difference in the mean age of disease onset was statistically significant between genders (30.2 ± 14.5 years in males and 27.1 ± 15.6 in females, *P* < 0.05). And 52.3% of all patients had onset before 40 years old. There were significant differences between males and females in the distribution sites of lesions (See Table 4). Furthermore, 82.8% of patients suffered from pruritus, and among them 9.1% developed sleep disturbances and 26.9% had scratches.

**Table 3:** Clinical features and treatment history of patients

Characteristics	No. of cases (%)
Total	*N* = 12031
**Treatment history**	
** Systemic treatments**	9600 (79.8)
TCM*^a^*	8661 (72.0)
Vitamin A derivatives	2604 (21.6)
Immune Depressants	1297 (10.8)
Glucocorticoids	1577 (13.1)
** Topical treatments**	10787 (89.7)
Glucocorticoids	9113 (75.7)
Vitamin D3 analog	3634 (30.2)
Tazarotene	2976 (24.7)
TCM*^a^*	2802 (23.3)
Salicylic Acid	2295 (19.1)
Other Therapy	2634 (21.9)
Photo Therapy	2220 (18.5)
Hydro Therapy	575 (4.8)
**Phenotypes**	
** Psoriasis vulgaris**	11606 (96.5)
** Guttate type**	3503 (29.1)
plaque type	6147 (51.1)
mixed type	1956 (16.3)
**Pustular psoriasis**	189 (1.6)
palms and soles type	41 (0.3)
generalized type	148 (1.2)
**Erythrodermic psoriasis**	83 (0.7)
**Psoriatic arthritis*^b^***	153 (1.3)

*a*, traditional Chinese medicine.

*b*, obtained from the patients history on a questionnaire.

**Table 4:** Comparison of lesions distribution and triggering factors between genders

	Total *N* = 12031 *n*(% = *n*/*N*)	Male (*N*_1_ = 7206) *n*_1_(% = *n*_1_/*N*_1_)	Female (*N*_2_= 4825) *n*_2_(% = *n*_2_/*N*_2_)	*p*
Triggering factors				
Season	7243 (60.2)	4388 (60.9)	2861 (59.3)	0.203
stress	4151 (34.5)	2414 (33.5)	1790 (37.1)	< 0.001
Smoking	626 (5.2)	533 (7.4)	92 (1.9)	< 0.001
Alcohol	2214 (18.4)	1794 (24.9)	405 (8.4)	< 0.001
Sphagitis	3296 (27.4)	1816 (25.2)	1486 (31.0)	< 0.001
Lesions distribution				
Leg	9348 (77.7)	5355 (74)	3993 (83)	< 0.001
Scalp	9024 (75.0)	5797 (80)	3227 (67)	< 0.001
Elbow	8069 (67.1)	4757 (66)	3312 (69)	0.183
Thigh	8042 (66.8)	4558 (63)	3484 (72)	< 0.001
Forearm	7620 (63.3)	4402 (61)	3218 (67)	< 0.05
Back	7602 (63.2)	4173 (58)	3429 (71)	< 0.001
Upper arm	7298 (60.7)	4104 (57)	3194 (66)	< 0.001
Waist	6639 (55.2)	3719 (52)	2920 (61)	< 0.001
Abdomen	6405 (53.2)	3442 (48)	2963 (61)	< 0.001
Knee	6293 (52.3)	3635 50)	2658 (61)	< 0.05
Bottom	5867 (48.8)	3303 (46)	2564 (55)	< 0.001
Chest	5782 (48.1)	3273 (45)	2509 (53)	< 0.001
Ear canal	3863 (32.1)	2374 (33)	1489 (52)	0.085
Palm	1244 (10.3)	733 (10)	511 (31)	0.505
Foot Plate	1170 (9.7)	667 (9)	503 (11)	0.055
perineum*^a^*	1145 (9.5)	680 (9)	465 (10)	0.738
Mucosa*^b^*	186 (1.5)	123 (2)	63 (1)	0.085
Glans	526 (4.4)	526 (7)		

Chi-square test, significant at *p* < 0.05.

*a*, include scrotum in men.

*b*, especially indicate lip mucosa.

### Phenotypes of psoriasis

Majority of patients (96.5%) were psoriasis vulgaris, 1.6% were pustular psoriasis, 0.7% were erythrodermic psoriasis, and 1.3% were Psoriatic arthritis (See Table 3). Among 11751 patients with non-missing values on disease activity, 43.3% were in active stage, 34.1% in stable, 15.3% in resolving, and 7.3% in remitting stage.

### Disease severity of the patients

The severity of our patients was evaluated on the basis of involved body surface area (BSA) and Psoriasis area and severity index (PASI) scoring method. The mean percentage of BSA that had skin lesions was 17.94 (SD20.06). Based on BSA involvement, 42.7% of patients were categorized as mild, 40.6% as moderate and 16.7% as severe. The mean PASI score was 18.70 (SD10.01), indicating that most patients attending hospitals had moderate-to-severe psoriasis (See Table 5).

**Table 5:** Disease severity based on the BSA and PASI score

	*N*	%
**Mean percentage of skin lesion on body surface area (SD)**	17.94 ± 20.06	
Mild	4884	42.7
Moderate	4652	40.6
Severe	1914	16.7
**Mean PASI scores of skin lesion (SD)**	18.70 ± 10.01	
Mild	1736	15.3
Moderate	1532	13.5
Severe	8047	71.1

BSA, body surface area, grouped by mild (< 10%), moderate (10%∼30%), severe (> 30%).

PASI, Psoriasis Area and Severity Index, grouped by mild (< 8), moderate (8∼12), severe (> 12).

### Comorbidities

Among patients who took the tests, the most common comorbidity was hypertension (16.4%), followed by hyperlipidemia (13.7%), diabetes (7.8%), and coronary heart disease (2.4%, See Table 6).

**Table 6:** Characteristics of disorders on nail, joint and metabolism of patients

Characteristics	*N* = 12031[% = *n*/*N*]
**Nail Damage *n* (%)**	3592 (29.9)
Nail pitting	2167 (18.)
Oil drops	1765 (14.7)
Subungual hyperkeratosis	1395 (11.6)
Longitudinal ridging of nails	1326 (11.0)
Onycholysis	856 (7.1)
Onychomadesis	300 (2.5)
**Joint Damages***^a^* ***n* (%)**	740 (6.2)
Digital joints	406 (3.4)
Knee joint	356 (3.0)
Toe joint	190 (1.6)
Ankle joint	131 (1.1)
Elbow Joint	114 (0.9)
Wrist joint	114 (0.9)
Vertebral column	94 (0.8)
**Joint Symptoms**	
Red	207 (1.7)
Swelling	311 (2.6)
Pain	588 (4.9)
Deformity	222 (0.3)
**Weight *n* (%)**	***N* = 10955**
Obese	574 (5.2)
**Clinical and Laboratory parameters abnormal *n* (%)**	
**Blood pressure**	***N* = 12031**
Abnormal *n* (%)	1976 (16.4)
**Liver Functional Test**	**N = 6286**
Abnormal n (%)	224 (3.6)
**Renal Functional Tests**	***N* = 6120**
Abnormal *n* (%)	91 (1.5)
**Lipid Test**	***N* = 6001**
Increased Blood lipid *n* (%)	824 (13.7)
**Blood Glucose Test**	***N* = 5978**
Abnormal *n* (%)	468 (7.8)

*a*, according to the Moll and Wright criteria, other joint damage is defined as redness and swelling, pain or deformation in a joint last for more than 1 month.

### Characteristics of nail changes, joint involvement

Nail changes were observed in 29.9% of psoriasis patients. Joint damages were observed in 6.2% of patients including 1.3% of patients with PsA (See Table 6).

### Factors causing relapse and/or aggravation

As shown in Table 4, the most frequently reported cause of relapse or aggravation was season change (60.2%), followed by psychological stress (34.5%), sphagitis (27.4%), dietary factors (23.7%), alcohol consumption (18.4%), medication (5.3%), and smoking (5.2%). Nearly half reports about weather as aggravating factor were related to winter season (48.8%), followed by spring (23.1%), autumn (17.1%), and summer (8.4%).

Aggravation of skin lesions due to sunlight exposure was reported by 11.6% of patients, whereas positive effects of sunlight were reported by 17.5% and no impact by 67.5% of patients. There was no significant difference of the impact of season change on relapse and aggravation between male and female. However, the impacts of mental stress, smoking, alcohol consumption and sphagitis were significantly different between genders (See Table 4). The longest mean time of remission was 15.46months (SD40.28). A history of isomorphic effect (Koebner's phenomenon) was found in 39.4% of patients.

## DISCUSSION

Up to now, only two population-based studies were conducted in China in 1984 and 2012, respectively [16, 17]. These two studies report a lower prevalence of 0.12% and 0.47% of psoriasis in China, but details of clinical feature are less described. Similarly, there is also lack of population-based detailed descriptive study on clinical characteristic of psoriasis patients all over the world. This manuscript report a nationwide study focusing on clinical features, phenotypes, comorbidities, and etc. in the Han Chinese population. As far as we know, this is the largest study on clinical features, triggering factors and comorbidities of Psoriasis among Han Chinese population.

In the study, the sex ratio was 1.49:1 which is consistent with findings of previous studies in China [16], while the sex ratio in German and UK was nearly 1:1. The difference could be due to that more male patients sought medical care for psoriasis. We found that female patients had disease onset at a significantly younger age than males, coincide with a recent study showing that median age at onset was 25 and 28 years among women and men respectively [18]. And over half had disease onset before the age of 40. Scalp was the most frequently reported onset site (52.8%), which is similar to studies in Japan (59.4%) and the USA (52%∼56%) [5, 19]. In this study, over 70% of patients had severe psoriasis assessed by PASI scores. Genetic basis is involved in psoriasis, and family history is one of the strongest risk factors. Studies in Caucasians showed that about 30% of patients had an affected first-degree relative and their first-and second-degree relatives had an increased risk of psoriasis [20]. The risk of psoriasis vulgaris was found to be two to three times greater in monozygotic than in dizygotic twins [17, 21]. In our study, over 20% of patients had positive family history, which was more frequent in first-degree relatives than in second- and third-degree relatives. This is consistent with previous

Consistent with the finding of the 1984 nationwide survey in China, we found that most Chinese patients suffered from psoriasis vulgaris (96.5%), of which plaque was the dominant type (51.1%) [16]. When assessed by BSA involvement, more patients were categorized as having moderate-to-severe psoriasis in our study (57.3%) than that in the 1984 nationwide survey (37.2) [16]. The difference may be partially explained by different study design and changes in environments and health-care seeking behaviors.

In our study, only 1.3% reported that they had ever been diagnosed with PsA. And joint damages were observed among 6.2% of all patients, which is lower than the prevalence (5.8%) found in another Chinese study. However, PsA prevalence is much higher in Caucasians and other Asian population like Japanese and Korean [8, 9]. This is probably due to the difference in genetic backgrounds and diagnostic criteria. On the other hand, since PsA was not diagnosed with serological tests and imaging examinations in our study, some early stage patients may be leave out, therefore, there might be an underestimate of PsA prevalence in the analyzed population.

In our study, over 80% of patients suffered from pruritus, which is similar to that among Italian psoriasis patients and much higher than German patients. High prevalence of pruritus found in our study represents great impact on the quality of life among psoriasis patients in China.

It is well known that a number of factors may trigger the onset and aggravation of psoriasis. Recent surveys from China have identified that infection, mental stress, medication, moisture, alcohol consumption, and smoking are important risk factors for psoriasis [2, 16, 23].

In the present study, the top three most frequently reported factors for relapse and aggravation were season (60.2%), mental stress (34.5%), and pharyngitis and tonsillitis (27.4%). The 1984 nationwide survey also found weather conditions (32. 9%), infections (22.4%) and mental factors (12.2%) to be the most frequent causes of psoriasis deterioration. Since aggravation most frequently appears in winter and spring, it is thought that the disease is related to the decrease in temperature, humidity and daylight in these seasons.

Mental stress was found to be a primary aggravation factor. In another survey of psoriasis patients in China, 60.8% and 10.6% of patients believed that they had social and career development problems directly related to psoriasis [17]. A population-based study showed that history of psoriasis was independently associated with increased risk of major depression among individuals with limited or extensive psoriasis [24]. These findings suggest that it is important to attend to patients’ mental stress and give psychological intervention. Much more people reported alcohol as aggravation factors in our survey than in the 1984 survey, which is probably owing to the remarkable changes in Chinese people's life style.

We also fount that women often reported mental stress and sphagitis as aggravation factors, while men reported smoking and alcohol more often. This suggests that physicians should pay attention to different factors and give appropriate advice when dealing with female and male patients.

Metabolic syndrome is a clinical syndrome characterized by a combination of multiple metabolic diseases. Recent studies from China and western countries have shown that patients with psoriasis had an increased prevalence of metabolic syndrome which may be linked to disease pathophysiology [3, 25, 26]. We found that 5.2% of psoriasis patients were obese, while latest data from a large registry (PSOLAR) in Caucasians showed that 80% of psoriasis patients were obese [26]. In our study, the prevalence of metabolic comorbidities were 10.2%, 5.9% and 4.3% respectively for hypertension, hyperlipidemia and diabetes, which is much lower than that found in France (26%, 27.5% and 11.0% for hypertension, hyperlipidemia and diabetes respectively) [27].

At last and notably, there were several limitations in our study. Firstly, due to objective conditions it is inappropriate and impractical to refer all psoriasis patients accepted serological and imaging examination to confirm the diagnosis of PsA, the questionnaire just inquiry about the joint symptoms and have ever been diagnosed with psoriatic arthritis. So we were unable to determine the accurate prevalence of PsA in the sample. Secondly, many patients were not willing to undergo blood tests so that we only analyzed a subset of the sample for the prevalence of metabolic diseases. The prevalence of metabolic diseases may be underestimated if more patients who were not tested had those metabolic conditions. Further studies are warranted to establish simple and precise PsA and metabolic disease evaluation system for clinical use.

In summary, our study was done with a large sample size. We found that there were many similarities in onset age, onset site, clinical phenotypes and aggravation factors between Han Chinese and Caucasian psoriasis patients. However, the prevalence of PsA and metabolic diseases were much lower among Chinese psoriasis patients than Caucasians which provides some insights into the impact of environmental and/or genetic factors. Moreover, since mental factors play a more and more important role in the induction of the disease, physicians should take patients′ mental status seriously in the process of medical activities.

## MATERIALS AND METHODS

### Participant recruitment and study procedure

Under the coordination of the Psoriasis Investigation Group of the China Dermatologist Association from November 2009 to June 2010, a nationwide survey of psoriasis patients was conducted at the department of dermatology of 56 hospitals in 33 cities across China. The stratified sampling method was used to select cities. Patients of Han ethnic group who were diagnosed with psoriasis by a dermatologist at participating hospitals were recruited in the study by convenience sampling method. After informed consent was obtained, patients who were willing to participate were enrolled and interviewed using a standard questionnaire. The following information was collected: demographics, family history, history of smoking and alcohol intake, prior treatments, disease status including onset sites, onset age, causes of relapse or flare episodes, systemic symptoms and concomitant conditions during the course of the disease. Medical history of hypertension, hyperlipidemia, diabetes and coronary heart disease that were diagnosed by physicians were also inquired. All patients underwent physical examinations including measurements of weight, height, waist circumference and blood pressure. Dermatological examinations were conducted to assess morphological subtypes (guttate, plaque, combined type psoriasis, pustular psoriasis, and erythrodermic psoriasis).

Body surface area (BSA) and psoriasis area severity index (PASI) were used to evaluate the disease severity. A visual inspection of oral mucosa, genitals and nails was also included.

For patients who were willing to give blood samples, tests for ALT, AST, blood urea nitrogen, serum creatinine, cholesterol, triglycerides, and fasting blood glucose performed. Prior to the survey, all physicians involved in the study at participating hospitals were trained according to the same training manual on case definition and conducting examinations. Physicians were required to check questionnaires for missed items and logical errors right after each interview and make revisions accordingly.

### Measures

Psoriasis activity at the time of presentation was graded according to patients’ self-assessment as active (continuous appearance of new lesions and no resolution of older ones), stable (persistence of existing lesions), resolving (improvement or disappearance of lesions), and remitting (almost none residual lesions).

Psoriatic arthritis incidence obtained from the patients history on a questionnaire According to Moll and Wright criteria, other joint damage is defined as redness and swelling, pain or deformation in a joint last for more than 1 month, confirmed by physical examination and medical records [19]. The diagnosis of metabolic comorbidities was mainly determined by medical history and examination in the survey. Abnormal metabolic values were defined as the following criteria: abdominal obesity (waist circumference > 102 cm in men and > 88 cm in women), hypertriglyceridemia (≥ 150 mg/dL), low levels of high-density lipoprotein cholesterol (< 40 mg/dL in men and < 50 mg/dL in women), high blood pressure (≥ 140/90 mm Hg), and high fasting glucose levels (≥ 100 mg/dL).

### Statistical analysis

A statistician who was not involved in data collection for the study performed statistical evaluation independently using SPSS17.0 software. All patients were included in the statistical analysis. Descriptive statistics, 2 tailed *t*-test and chi-square test were appropriately used for the analysis of the data.
